# Motor recovery monitoring using acceleration measurements in post acute stroke patients

**DOI:** 10.1186/1475-925X-12-33

**Published:** 2013-04-16

**Authors:** Jayavardhana Gubbi, Aravinda S Rao, Kun Fang, Bernard Yan, Marimuthu Palaniswami

**Affiliations:** 1 Department of Electrical and Electronic Engineering, The University of Melbourne, Parkville, Melbourne, VIC - 3010, Australia; 2 Department of Neurology, Huashan Hospital, Fudan University, Shanghai 200040, China; 3 NeuroIntervention Service, Department of Radiology, The Royal Melbourne Hospital, Grattan Street, Parkville, Melbourne, Victoria 3050, Australia

## Abstract

**Background:**

Stroke is one of the major causes of morbidity and mortality. Its recovery and treatment depends on close clinical monitoring by a clinician especially during the first few hours after the onset of stroke. Patients who do not exhibit early motor recovery post thrombolysis may benefit from more aggressive treatment.

**Method:**

A novel approach for monitoring stroke during the first few hours after the onset of stroke using a wireless accelerometer based motor activity monitoring system is developed. It monitors the motor activity by measuring the acceleration of the arms in three axes. In the presented proof of concept study, the measured acceleration data is transferred wirelessly using iMote2 platform to the base station that is equipped with an online algorithm capable of calculating an index equivalent to the National Institute of Health Stroke Score (NIHSS) motor index. The system is developed by collecting data from 15 patients.

**Results:**

We have successfully demonstrated an end-to-end stroke monitoring system reporting an accuracy of calculating stroke index of more than 80%, highest Cohen’s overall agreement of 0.91 (with excellent *κ* coefficient of 0.76).

**Conclusion:**

A wireless accelerometer based ‘hot stroke’ monitoring system is developed to monitor the motor recovery in acute-stroke patients. It has been shown to monitor stroke patients continuously, which has not been possible so far with high reliability.

## Background

Stroke is a major cause of morbidity and mortality in Australia. There is an annual incidence of 48,000 new strokes and the risk of death is 25 to 30% [1]. Of those who survive, stroke contributes to 25% of all chronic disabilities in Australia. Acute stroke is caused by a blockage of one of the arteries in the brain resulting in interrupted blood supply. Brain cells deprived of oxygenated blood die rapidly unless blood supply is restored. One of the milestones of modern management of acute stroke is the administration of a thrombolytic (clot-busting medication) to unblock the blocked artery [2]. This restores blood supply to the brain and arresting the demise of brain cells. International multi-center studies have shown that the patients who receive thrombolytics have better clinical outcomes [2].

The delivery of thrombolytic agents to acute stroke patients requires round-the-clock availability of a stroke neurologist to clinically assess the patient. This is a critical step as there are dangerous mimics of stroke and a wrong diagnosis by a non-specialist will significantly impact upon the correct management decision. A recent survey by the National Stroke Foundation reported that 72% of Australian hospitals were unable to provide acute stroke treatment which results in deprivation of evidence-based standard stroke care for a significant proportion of stroke patients in rural Victoria. This translates to missed treatment opportunities in decreasing the morbidity and mortality associated with acute stroke [3]. In addition, the monitoring of motor recovery by clinical observation is critical in the management of stroke patients. Patients who do not exhibit early motor recovery post thrombolysis may benefit from more aggressive treatment. However, the current clinical observation paradigm is time-consuming and subjected to inter-observer bias. It follows that a portable device for continuous monitoring of motor recovery in stroke patients treated with thrombolysis, would signify a major advance in patient management.

Recent technological advances in low power integrated circuits and wireless communications have made available efficient, low cost, low power miniature devices for use in wireless sensing applications. Automated clinical decision making is one of the key research areas in biomedical engineering. A wearable body area network is a viable solution for the unhindered monitoring of patient condition [4]. Automatic patient monitoring systems (PMS) send an alert to the care giver based on the physiological parameters gathered. Wireless Body Area Network (WBAN) is one of the key emerging technologies for unobstructive health monitoring [5]. In an editorial overview about wearable systems by Bonato [6] published in 2003, he emphasizes the impact of technological breakthrough in sensors and sensor networks on biomedical engineering. His observations are indeed true eight years on and we have seen unhindered physiological monitoring using wearable wireless systems.

Assessment of the effect of thrombolysis is the core motivation to develop an automated monitoring tool for the assessment of post-stroke individuals’ during the ‘hot’ period after stroke (while the patient is still in the hospital). The National Institute of Health Stroke Scale (NIHSS) is an international initiative to systematically assess stroke and provide a quantitative measure of all stroke related neurological deficit. It is used as a clinical assessment tool to evaluate acuity in stroke patients, determine appropriate treatment and predict patient outcome. It has been proven to be a very useful tool in treatment planning by Neuro-intervention for acute stroke patients. The scale is a 17-item neurological examination to evaluate the levels of consciousness, language, neglect, visual-field loss, extraocular movement, motor strength, ataxia, dysarthria, and sensory loss. The score ranges from 0 to 42. The single patient assessment requires about 10 minutes to complete and is performed every hour by a qualified doctor. In our work, we are interested in motor strength assessment which is defined as in Table 1[7].

**Table 1:** **NIH Stroke Scale description for motor activity analysis [**[7]**]**

**Scale**	**Status**	**Description**
0	No drift	Limb holds 90 degrees for a full 10 seconds
1	Drift	Limb holds 90 degrees but drifts down before full 10 seconds
2	Some effort against gravity	Limb cannot get to 90 degrees, but has some effort against gravity
3	No effort against gravity	No effort against gravity
4	No movement	-
UN	Amputation	-

Major efforts involving accelerometer have been in activity monitoring as a fitness aid using smart phones as the base platform. Accelerometer sensors with gyroscope have been used in such fitness products now commercially available. A good summary of the work using these sensors in activity monitoring can be found in [8] including a comparison of commercially available system. Bouten et. al. [9] developed a basic activity monitoring system using triaxial accelerometer and proved the correlation of energy expenditure with processed accelerometer sensor signal was high on healthy subjects. Since then, various application areas where movement monitoring is required has been impacted. Often, the areas of focus have been in rehabilitation which is a reactive response rather than a proactive response. Nevertheless, these technological advances have resulted in the assessments by health care providers becoming more objective. In a detailed study, Yang *et. al.*[10] have proposed a neural classifier for activity recognition using accelerometer data. Their focus is mainly for signal processing and pattern recognition aspects and they classify eight daily activities on seven subjects with over 95% accuracy. In 2009, Roy*et. al.*[11] proposed a combined accelerometer-sEMG system for monitoring post stroke patients. They conduct their tests on 10 subjects who had stroke onset between 7±6 years before the test. They use 11 tasks for identifying the functional independence with 95% sensitivity using a hybrid neural classifier. It should be noted that the post-stroke in their work refers to period after several years of stroke.

Another area of research in post stroke assessment using accelerometer sensors is conducting Wolf Motor Function Test (WMFT) [12].WMFT is a post stroke assessment procedure carried out within days after the onset of stroke. It is a time based method to evaluate upper extremity performance in chronic stroke patients. Parnandi *et. al.*[13,14] have developed a wireless accelerometer system which replicates WMFT conducted by trained personnel. The assessment is based on 15 tasks rated according to time and quality of motion. They compare the scores obtained from their proposed method with therapist’s scores and report an average error of 0.0667 which is excellent. Patel *et. al.*[15] propose a similar system to quantify the Functional Ability Scale (FAS) score in stroke survivors using three sensors with excellent outcomes. In another interesting work by Lopez-Meyer *et. al.*[16], assessment of rehabilitation FAS scores using gait is proposed with the sensors embedded in the shoes to monitor the gait. Once again, [13,15]**,**[16] are in post stroke assessment spanning days after the onset of stroke and falls under the post stroke rehabilitation category.

Unlike other methods proposed in the literature, our work focuses on post stroke monitoring during ‘hot’ hours which is usually the first 24 hours after the onset of stroke. In this paper, we propose to develop a wireless accelerometer based system in order to monitor the motor recovery in acute-stroke patients in the hot hour. Accelerometer data obtained from these sensors will be used to develop a new algorithm for stroke monitoring and patient recovery.

## Method

**Figure 1:**
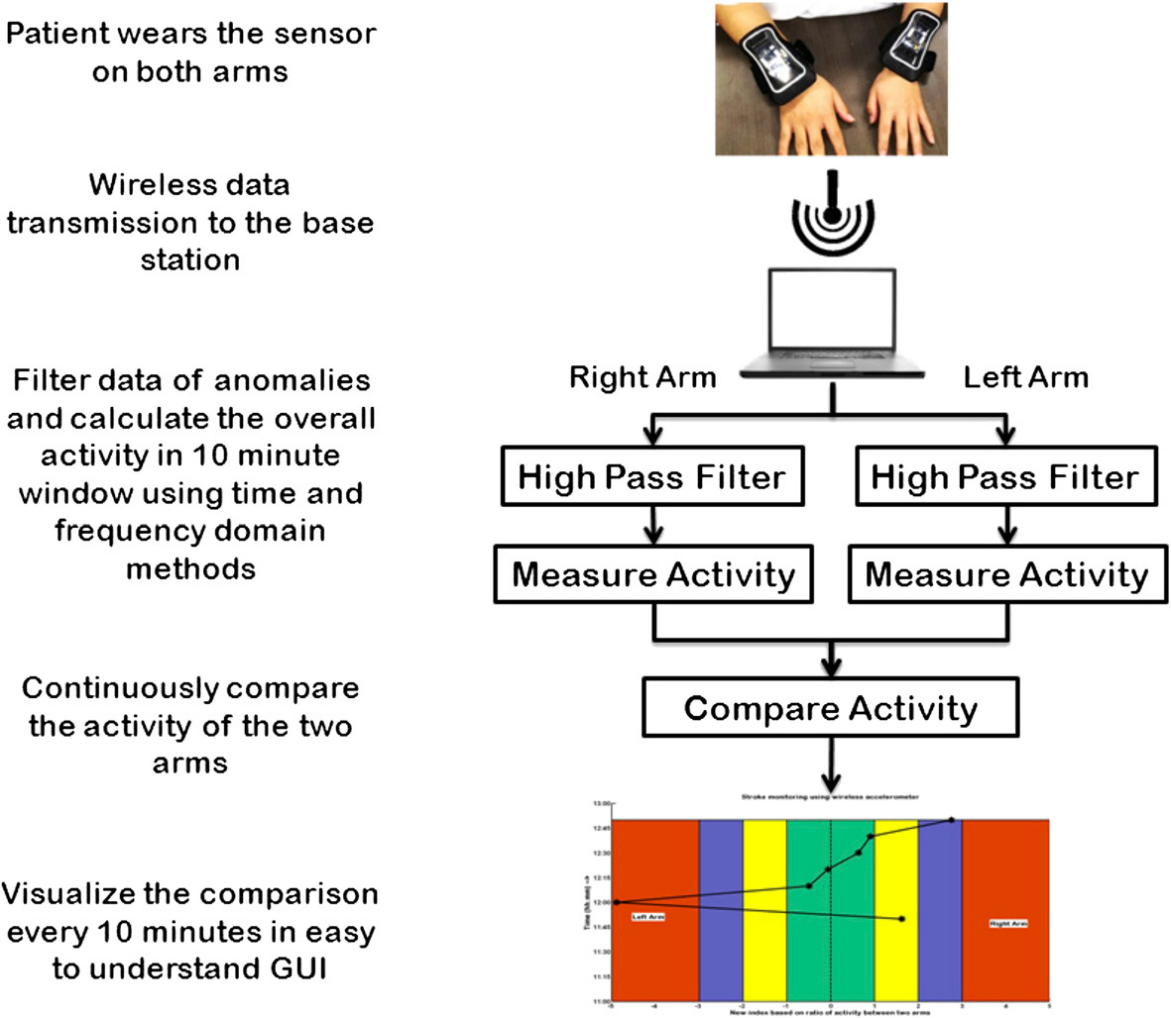
Schematic of the overall methodology describing the steps from sensor data collection to visualization.

We develop a new system which when put on patients’ arms will monitor the motor activity continuously. Further, we correlate the scores generated by the developed automated algorithm with an expert score at motor recovery band in NIHSS [7]. The observed indices are retained as in Table 1. However, as per the definition of NIHSS indices 3 and 4, motor activity monitoring is virtually impossible using an accelerometer and hence we have represented 3 and 4 as 3 for all our analysis. The schematic of the proposed method is summarised in Figure 1 and briefly the procedure is as follows. The data is collected using a wireless sensor node attached to a 3-axis accelerometer. The data collected at a predetermined sampling rate is transmitted from both the arms to the base station. On the base station, the data is pre-processed using a basic high pass filter and the activity in a 10 minute window is calculated. The activities of the two arms are compared and visualized in a custom designed graphic user interface.

### Wireless accelerometer sensor data acquisition

At this stage of our experimentation, we use Crossbow iMote2 as the sensor platform for collecting the acceleration data. The Imote2.NET is an advanced wireless sensor node platform. It is built around the low-power PXA271 XScale CPU and also integrates an 802.15.4 compliant radio at 2.4*GHz*. The design is modular and stackable with interface connectors for expansion boards on both the top and bottom sides. The top connectors provide a standard set of I/O signals for basic expansion boards and we use ITS400 sensor board. It contains a three-axis accelerometer, an advanced temperature/humidity sensor, a light sensor and a 4 channel A/D converter out of which we use tri-axial accelerometer in our experiments. The sensors are mounted on each arm of the patient using an armband and the sensor readings are transferred wirelessly to an iMote2 base station which is connected to a computer via USB connection. This enables the patient to move freely in a given perimeter. Considering the patient is affected by stroke and the measurement is taken within the first 24 hours, movement is usually limited and confined to the wards in any clinical setup. The data is stored using a MySQL server running locally and MATLAB is used for *online* analysis and data visualization. The sensitivity of the on board ST accelerometer is ±2*g*.

### Data collection and preprocessing

**Figure 2:**
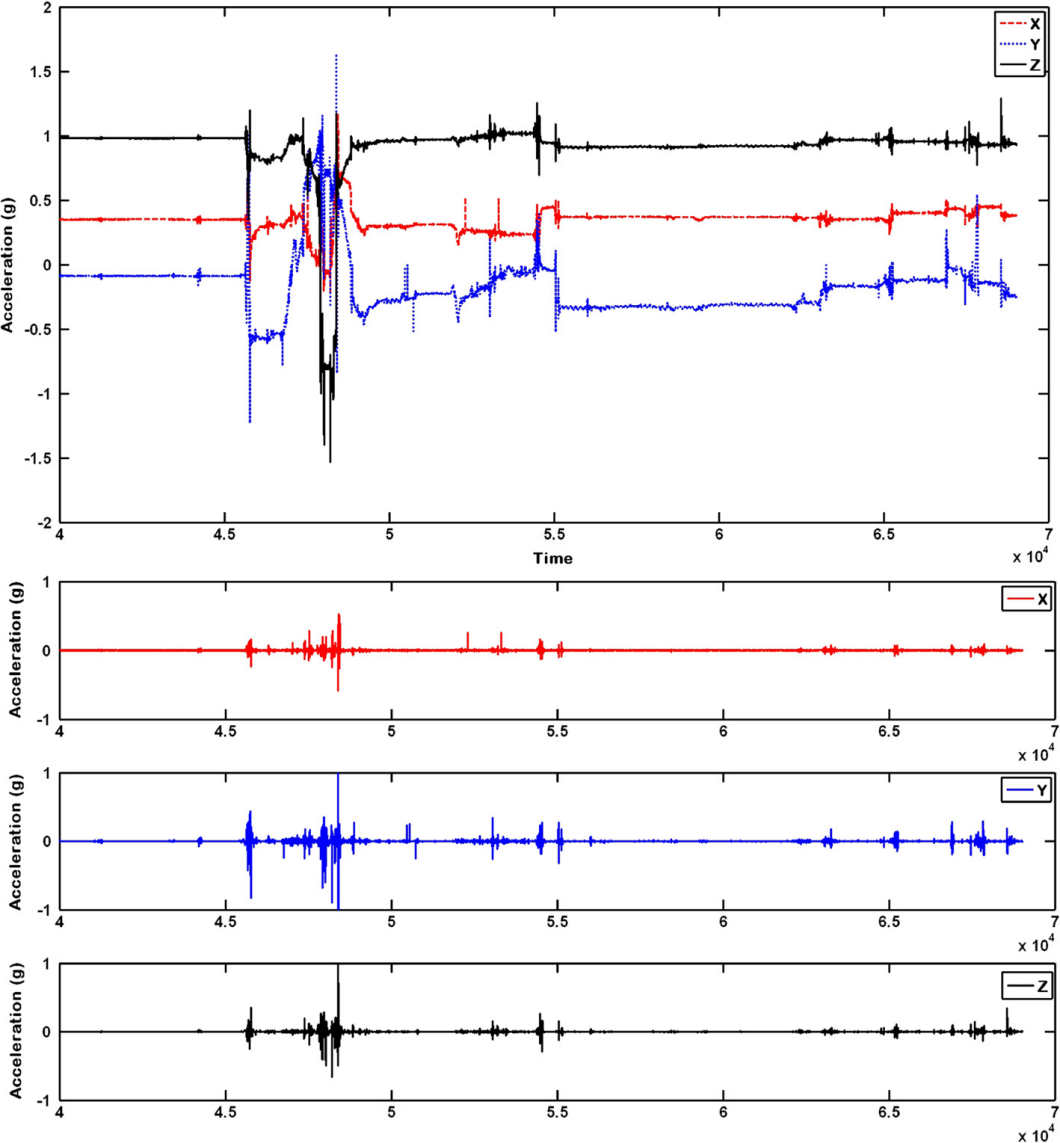
**Raw accelerometer readings and filtered outputs.** Raw accelerometer readings of a patient’s affected hand: Top - Original received from iMote2; Bottom - Filtered signal.

The movement data was collected in Melbourne Brain Centre, Royal Melbourne Hospital, Australia. The research protocol was approved by Royal Melbourne Hospital Human Research Ethics Committee (2010.245). In total, 25 subjects were used to develop the method which included 15 acute stroke patients (8 males, 7 females) and 10 controls (2 males, 9 females). The average age of patients was 69.8±15*years* and the average age of controls was 60±16*years*. In the algorithm presented, the data from 10 controls was not utilized. The summary of the patient data including age, sex, diabetes, smoking and hypertension is given in Table 2. The accelerometer data was collected for the first four hours and another one hour after 24 hours. An expert neurologist records the observed NIHSS motor scores and the observed NIHSS overall score at the time of onset ( 0*th* hour), 1*st* hour, 2*nd* hour, 3*rd* hour and at 24 hours. For instance, if the patient comes to the hospital at 9*am*, the data is collected between 9*am* and 1*pm* on that day (expert recording at 9*am*,10*am*,11*am*,12*pm*) and between 9*am* and 10*am* on the following day (expert recording at 9*am* on the following day). The sampling frequency of the data collected is 100 Hz and three packets of data transfers to the base station every second. Each packet received contained several values of acceleration values along *x*, *y*, and *z* axes. The time stamp was generated at the base station instead of the source node in order to avoid time synchronisation problems in wireless sensor nodes [17]**]. In total, six acceleration values were received at any given instant (2 arms × 3 axis). This signal was filtered using a Butterworth 6**^*th*^ order high-pass filter with 1 Hz as cutoff frequency. The original raw signal and the filtered output are shown in Figure 2.

**Table 2:** Summary of the patient data collected

**Patient details**						**Total data**		**Affected**	**NIHSS Score**				**NIHSS Score**			
						**collected**		**arm**	**(Overall)**				**(Motor)**			
**Sl. No.**	**Age**	**Sex**	**Diabetic**	**Smoking**	**Hypersensitive**	**Stage1**	**Stage2**		**T0**	**T1**	**T2**	**T24**	**T0**	**T1**	**T2**	**T24**
						**(Minutes)**	**(Minutes)**									
1	87	Male	No	No	Yes	183	60	Left	1	1	0	0	1	1	0	0
2	59	Male	No	No	Yes	161	69	Left	3	3	3	3	1	1	1	1
4	44	Male	Yes	Yes	No	131	26	Left	6	6	6	6	3	3	3	3
5	47	Male	No	No	No	218	83	Left	3	3	3	3	2	2	2	2
8	61	Male	No	No	Yes	165	68	Right	2	2	2	2	1	1	1	1
9	81	Female	Yes	No	Yes	161	72	Left	12	12	12	12	3	3	3	3
10	88	Female	No	No	Yes	162	×	Right	2	2	2	×	2	2	2	×
12	78	Female	Yes	No	Yes	162	90	Left	10	10	10	10	1	1	1	1
13	52	Female	No	No	No	161	128	Left	9	9	9	9	3	3	3	3
15	59	Female	No	Yes	No	82	66	Right	12	12	12	12	3	3	3	3
16	81	Female	No	No	Yes	82	68	Right	6	6	6	5	1	1	1	1
17	85	Female	No	No	No	83	70	Left	16	16	16	16	3	3	3	3
18	76	Male	No	No	Yes	91	60	Right	9	9	8	8	3	3	3	3
19	81	Male	No	No	No	80	75	Right	5	5	5	5	3	3	3	3
20	69	Male	No	No	Yes	80	77	Left	6	6	6	5	2	2	2	1

## Signal analysis and calculation of stroke index

In our experiments, we are interested in relative overall motion of the arms. We derive a score which is equivalent to the NIHSS motor activity and show that it is correlated to the observed NIHSS score. During the calculation of NIHSS score, an NIHSS accredited clinician calculates the score every hour and the test would take approximately 10 minutes. This motivates us to use a 10 minute window for calculating the motor activity. The idea is to calculate the activity of both arms every 10 minutes and compare the calculated activity and arrive at a meaningful index, which correlates with the observed NIHSS motor score. In order to achieve this, three standard acceleration data analysis techniques were employed. Ambrosini *et. al.*[18] propose a new design of a symmetry controller for cycling induced by functional electrical stimulation and test it on post-acute stroke patients (rehabilitation phase). Making use of the cyclical nature of pedalling in their experiment, they design a new algorithm for balancing the electrical stimulation in order to recover motor recovery. Their symmetry index is based on the cyclical activities of the two limbs. However, in the case of ‘hot stroke’, which we are dealing with, continuous activity cannot be ensured and a more generalised method is required for calculating the imbalance. In this paper, we have developed three very basic schemes for calculating such imbalance and the summary of the methods used in the development of the algorithm is given in Table 3.

**Table 3:** Summary of methods employed in signal analysis

	**Norm based**	**Signal Magnitude Area based**	**Energy based**
Domain	Time	Time	Frequency
Metric	Euclidean distance	Manhattan distance	Euclidean distance
Activity measured	Cumulative velocity	Cumulative velocity of	Energy of activity
		average activity in *x*, *y*, and	in a 10 minute window
		*z* directions	
Calculation method	Every sample	Average value over 10 minutes	Window of 10 minutes
Sensitivity	Low	High for normal patients and Low	High
		for severely disabled patients	
Computational	Low	Low	High
complexity			

### Norm based index

In this method, average activity of each arm is calculated and the Euclidean distance between the activities is used for deriving the index. We first calculate the magnitude of the three dimensions at every instance using equation 1.

(1) $$A_{i}=\sqrt{{\left(x_{i}\right)}^{2}+{\left(y_{i}\right)}^{2}+{\left(z_{i}\right)}^{2}}$$

where, *A*_*i*_ is the resultant acceleration magnitude (calculated at every instance) using the accelerations along *x*, *y* and *z* axes for both the arms. In the second stage, a 10 minute window is considered and a cumulative integral is calculated to obtain velocities. The area under the velocity curve represented by *R*_*N*_ and *L*_*N*_ are calculated every 10 minutes (results in a scalar quantity for every 10 minute window). The ratio of the velocities indicates the activity of any arm against the other. In order to maintain the ratio comparable and for detecting the defective (paralysed) arm, we use equation 2.

(2) $$S_{N}=\left\{\begin{array}{ll} \frac{L_{N}}{{\left(L_{N}+R_{N}\right)}^{2}} & \text{if}\;L_{N}>R_{N}\\ \frac{R_{N}}{{\left(L_{N}+R_{N}\right)}^{2}} & \text{if}\;L_{N}<=R_{N} \end{array}\right)$$

where, *S*_*N*_ is the norm based stroke index. If *L*_*N*_>>*R*_*N*_, it implies paralysed right arm and *vice versa*.

### Signal Magnitude Area (SMA) based index

Signal Magnitude Area is defined as the sum of acceleration magnitude summations over three axes normalized by the length of the signal. It reflects the activity within a time window instead of at every instance. This has been successfully used in activity recognition by Yang *et. al.*[10] as a feature and we use it to measure relative activity between the two arms in a time window. It is analogous to the city block distance and is given by the equation 3.

(3) $$\mathrm{SM}A_{j}=\frac{1}{w}\left(\overset{w}{\underset{i=0}{\sum }}|x_{i}|+\overset{w}{\underset{i=0}{\sum }}|y_{i}|+\overset{w}{\underset{i=0}{\sum }}|z_{i}|\right)$$

where *j* indicates the window number with length *w*. In our experiments, we have empirically chosen the window length as one second. Similar to norm based method above, a 10 minute window is considered in the second stage and a cumulative integral is calculated. The area under the curve ( *L*_*S*_ and *R*_*S*_) is calculated every 10 minutes. The ratio of the integrals is calculated for relative arm activity. In order to maintain the ratio comparable and for detecting the defective (paralyzed) arm, we use equation 4.

(4) $$S_{S}=\left\{\begin{array}{ll} \frac{L_{S}}{{\left(L_{S}+R_{S}\right)}^{2}} & \text{if}\;L_{S}>R_{S}\\ \frac{R_{S}}{{\left(L_{S}+R_{S}\right)}^{2}} & \text{if}\;R_{S}>=L_{S} \end{array}\right)$$

where, *S*_*S*_ is the SMA based stroke index. If *L*_*S*_>>*R*_*S*_, it implies defective right arm and *vice versa*. It should be noted that SMA is identical to Manhattan norm of the accelerometer values.

### Average energy comparison based index

The frequency distribution in the affected and the unaffected arm are compared in this method. Preliminary work on using sensor networks for activity recognition was carried out by Wang *et. al.*[19] in activity recognition. Very similar to their work, the energy is calculated as the sum of squared FFT magnitude of the signal. The average of the three energy signals over a window of length one second is used in the calculation of the index. Similar to other methods described above, a 10 minute window is considered in the second stage and a cumulative integral is calculated within the window. The area under the curve ( *L*_*E*_ and *R*_*E*_) is calculated every 10 minutes. The ratio of energies indicates the activity of one arm against the other. In order to maintain the ratio comparable and for detecting the defective (paralysed) arm, we use equation 5.

(5) $$S_{E}=\left\{\begin{array}{ll} \frac{L_{E}}{{\left(L_{E}+R_{E}\right)}^{2}} & \text{if}\;L_{E}>R_{E}\\ \frac{R_{E}}{{\left(L_{E}+R_{E}\right)}^{2}} & \text{if}\;R_{E}>=L_{E} \end{array}\right)$$

where, *S*_*E*_ is the Energy in frequency domain based stroke index. If *L*_*E*_>>*R*_*E*_, it implies defective right arm and *vice versa*.

### Conversion to stroke index

**Figure 3:**
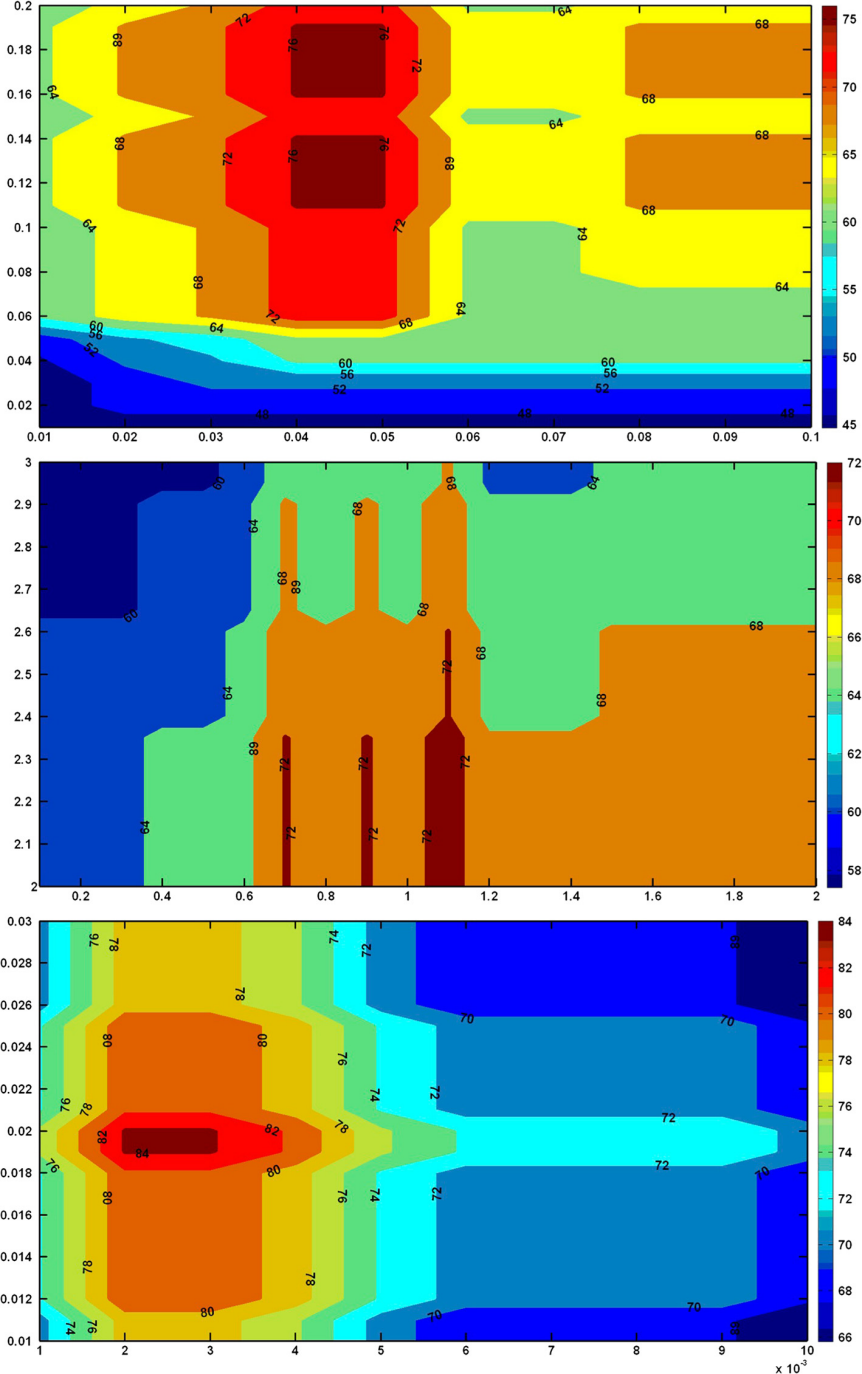
**Colormap to help choosing the thresholds.** Colour map for choosing appropriate thresholds for converting the accelerometer readings to equivalent motor index: Norm based (top), SMA based (middle), Energy based (bottom). The colormap shows the accuracy contour while x and y axes represent the two thresholds. As it can be seen, for certain values of calculated thresholds the accuracy is quite high (in red or maroon colors) particularly for the energy based method. This chart is obtained using *T*_0_, *T*_1_ and *T*_2_ values and is tested using *T*_24_.

The *S*_*N*_, *S*_*S*_ and *S*_*E*_ calculated above are the values which correlate with the observed NIHSS values. However, the values cannot be interpreted by the doctor. Hence we have devised a conversion method to convert these values to NIHSS scores. Two thresholds *Th*_1_ and *Th*_**2**_ have to be calculated to convert the indices into discrete values for easy comparison with NIHSS index. Neurology assessment by doctors with NIHSS accreditation has shown fair inter-rater agreement. Hence we define accuracy for the proposed system as the percentage of agreement between the scores given by a NIHSS accredited doctor and the proposed solution. For analysis, we have calculated the percentage accuracy of the system for different values of *Th*_1_ and *Th*_2_. The plot of the analysis using the three techniques is shown in Figure 3. The colour map shows the accuracy contour while x and y axes represent the two thresholds. As it can be seen, for certain values of calculated thresholds the accuracy is quite high (in red or maroon colours) particularly for the energy based method. This chart is obtained using *T*_0_, *T*_1_ and *T*_2_ values and is tested using *T*_24_.

### Visualisation

**Figure 4:**
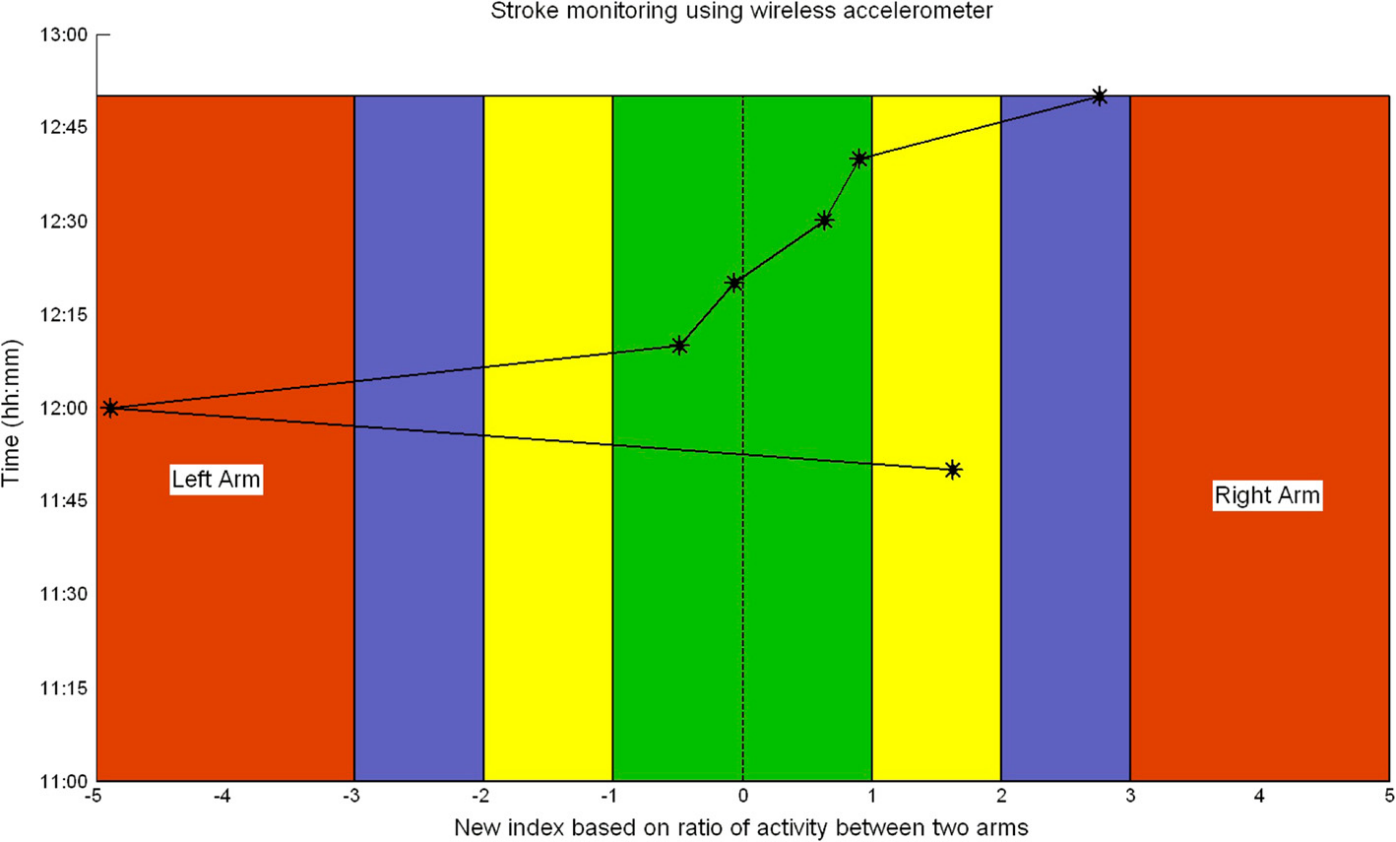
**Generic visualisation for stroke monitoring.** The graph is divided into two sections representing two arms. The colour coding is based on the severity of the stroke - green represents normal activity and red represents severe arm disability. The y axis represents time and x axis represents calculated stroke index. The figure is divided into two parts vertically along midway, indicating two arms. As we move away from 0 in either direction, the severity of stroke increases. The custom colour coding is based on the NIHSS stroke scale given in Table 1. The recording started at 11:45*am* and continued until 12:45*pm*. The doctor records only once during this period at the start of the recording (which is onset +24 hours) but the system records it continuously. Calculating the index within a 10 minute window has resulted in 7 discrete motor activity assessment. This is an evolving graph which gets updated every 10 minutes when the data collection is in progress.

As the system will be used by doctors in a hospital environment, it is important to create an easily understandable visualisation of the indices as the data is being collected online. In the initial stage, the problematic arm is not known to the system. As the patient comes in, two iMote2s will be strapped to the patient’s arms as explained earlier. The online visualisation we have created for stroke monitoring is shown in Figure 4 for day 2 of data collection for clarity. The figure is divided into two parts vertically along the midway, indicating two arms. In the figure, x axis indicates stroke index and y axis indicates the time of recording. Negative stroke index is for left hand and positive stroke index is for right hand. As we move away from 0 in either direction, the severity of stroke increases. The custom colour coding is based on the NIHSS stroke scale given in Table 1. In Figure 4, the recording started at 11:45*am* and continued until 12:45*pm*. The doctor records only once during this period at the start of the recording (which is onset +24 hours). However, the system presented here continuously monitors the patient enabling a detailed study of motor recovery. Recording every 10 minutes has resulted in 7 discrete motor activity assessment. This is an online (evolving) graph which gets updated every 10 minutes when the data collection is in progress. In the following section, we present results using the proposed methods and correlate it with expert’s observation.

## Results and discussion

The overall results of the developed method for 15 patients is shown in Table 4. Firstly, we discuss the prediction of the affected arm. Based on continuous arm activity, we keep track of the overall arm movement in order to predict the stroke affected arm. As it can be seen from Table 4, an accuracy of 93.33%, 93.33% and 100% is obtained for norm based, SMA based and energy based methods respectively.

**Table 4:** Results of stroke index computation using proposed methods

				**Observed**					**Norm based**					**SMA based**					**Energy based**			
**Patient**	**Day 1**	**Day 2**	**Affected**	**T0**	**T1**	**T2**	**T24**	**Affected**	**T0**	**T1**	**T2**	**T24**	**Affected**	**T0**	**T1**	**T2**	**T24**	**Affected**	**T0**	**T1**	**T2**	**T24**
**No.**	**(Mins)**	**(Mins)**	**Arm**					**Arm**					**Arm**					**Arm**				
1	183	60	Left	1	1	0	0	Left	1	1	**2**	**1**	Left	1	1	**2**	**1**	Left	1	**2**	**2**	**1**
2	161	69	Left	1	1	1	1	Left	1	1	1	1	**Right**	1	1	1	1	Left	1	1	1	1
4	131	26	Left	3	3	3	3	Left	3	3	3	3	Left	3	3	3	3	Left	3	3	3	3
5	218	83	Left	2	2	2	2	Left	**1**	2	2	**1**	Left	**1**	2	**1**	2	Left	2	2	2	2
8	165	68	Right	1	1	1	1	Right	**3**	1	1	1	Right	**3**	1	1	**2**	Right	**3**	**3**	1	1
9	161	72	Left	3	3	3	3	Left	3	3	3	3	Left	3	3	3	3	Left	3	3	3	3
10	162	×	Right	2	2	2	×	Right	2	**3**	2	×	Right	2	**3**	**1**	×	Right	2	2	2	×
12	162	90	Left	1	1	1	1	Left	**3**	1	1	**3**	Left	**3**	1	1	**3**	Left	**2**	1	1	**3**
13	161	128	Left	3	3	3	3	Left	3	3	**1**	**2**	Left	3	3	3	3	Left	3	3	3	3
15	82	66	Right	3	3	3	3	**Left**	3	×	×	**1**	Right	3	×	×	3	Right	3	×	×	3
16	82	68	Right	1	1	1	1	Right	1	**2**	×	**2**	Right	1	**2**	×	**3**	Right	1	**2**	×	**2**
17	83	70	Left	3	3	3	3	Left	3	3	×	3	Left	3	3	×	**2**	Left	3	3	×	3
18	91	60	Right	3	3	3	3	Right	3	1	×	3	Right	3	3	×	3	Right	3	3	×	3
19	80	75	Right	3	3	3	3	Right	3	3	×	3	Right	3	3	×	3	Right	3	3	×	3
20	80	77	Left	2	2	2	1	Left	2	**1**	×	1	Left	2	**1**	×	**3**	Left	2	2	×	**2**
Accuracy								93.33%	71.15%				93.33%	73.08%				100%	80.77%			

The first three columns give basic information about the recording. *T*_0_, *T*_1_ and *T*_2_ are recorded on the first day at the time of onset and every hour subsequent to that. *T*_24_ is recorded on the second day at *T*_0_+24 hours.

The observed values and calculated values using the three methods is given. × means, the recording was unavailable due to either recording technicians fault or fault in the hardware. The errors are highlighted.

Secondly, the observed NIHSS indices as well as the calculated values using the three proposed methods are presented. Under each category, *T*_0_, *T*_1_, *T*_2_ and *T*_24_ are given. ‘ ×’ in the table indicates that the values are not measurable (data unavailable status). During observation, if the patient is moved out of the ward for any reason, the values are not recorded and this is recorded as unavailable. Similarly, as iMote2 is a research prototype, battery and other communication errors lead to errors in the received signal. Based on the received time stamp the program automatically assigns an unavailable status. The accuracies are calculated considering only the available values and an accuracy of 80.77*%*,73.08*%* and 71.15*%* are obtained using energy based, SMA based and norm based methods respectively. It should also be noted that energy based technique results in over 70% accuracy in *T*_24_ prediction which are not used in calculation of the thresholds. The energy based method consistently results in standard deviation of 1 among the misclassified output as against the norm based and SMA based approaches.

Although the highest index prediction accuracy using energy based method appears low ( 80.77*%*), it should be noted that this implies 4 out of 5 indices are calculated perfectly and one index is varying just by standard deviation of 1. Moreover, the accelerometer based approach results in motor activity index in a much higher resolution of six readings every hour as against one reading every hour. The correlation of variables of calculated indices against the observed values has been assessed by the experts and they are accurate. Although this cannot be verified quantitatively due to resolution mismatch between the data, the experts have closely observed and agreed with the calculated index.

It is important to evaluate the results at varying severities. Table 6 shows the confusion matrix using the three methods. Rows indicate observed values and columns indicate accelerometer based predictions. As it can be seen clearly, energy based method outperforms norm based and the SMA based methods. It results in zero error for higher observed values of severity (2 and 3). However, if the observed value is low (1), the accuracy is only 56% with 27% being classified as medium severity (index 2) and the remaining as high severity (index 3). Non-linear signal processing techniques or more sophisticated features may be required to calculate these indices more accurately when the severity is very low. In order to validate our observations, we calculated Cohen’s statistics and the results are summarised in Table 5. Cohen’s index ( *κ*) is used as a measure to characterise the reliability of a computer program to match the expert observation and the ability of the method to reproduce the disease classifications [20]. Overall agreement score ( *p*_*o*_) and fair-chance corrected agreement ( *κ*) are calculated for the three proposed methods. Due to multiple levels of stroke index, we have also calculated weighted (linear and quadratic) and unweighted *κ* scores to reduce category bias on *κ*. As per our observations, the quadratic *κ* for observer-energy category results in and excellent overall agreement ( *p*_*o*_) of 0.91 and a fair-chance corrected agreement ( *κ*) of 0.76 ( *κ*>0.75 is considered excellent [21]).

**Table 5:** Confusion matrix showing the misclassification and the results as per three levels of severity- Norm based (left), SMA based (middle) and Energy based (right)

	**1**	**2**	**3**		**1**	**2**	**3**		**1**	**2**	**3**
1	13	2	3	1	11	2	5	1	10	5	3
2	3	5	1	2	4	4	1	2	0	9	0
3	3	1	19	3	0	1	22	3	0	0	23

Rows indicate observed indices.

**Table 6:** Cohen’s statistics for the three proposed methods

	**Unweighted**		**Linear**		**Quadratic**	
**Categories**	***p***_***o***_	***κ***	***p***_***o***_	***κ***	***p***_***o***_	***κ***
Observed-Norm	0.74	0.58	0.81	0.60	0.84	0.62
Observed-SMA	0.74	0.57	0.82	0.62	0.86	0.65
Observed-Energy	0.84	0.74	0.89	0.76	0.91	0.76

Comparison of Unweighted, Linear weighted and quadratic weighted *p*_*o*_ and *κ* calculated for the three methods.

The algorithm was implemented in MATLAB using a laptop with 4GB RAM and Intel i7 processor. The database used as MySQL and Microsoft C# was used for data collection and storing. The algorithm runs almost instantaneously and the first visualisation output occurs a few seconds after the first 10 minutes of data collection (as the analysis window chosen is 10 minutes).

The impact of this work is that it lays the foundation for the development of a device in the monitoring of neurological function post acute stroke. The management of acute stroke is time critical and resource intensive. Particularly important is the window period of 2 to 3 hours after thrombolysis whereby an opportunity exists to re-intervene in patients who fail to respond to the first treatment. Continuous monitoring of neurological recovery in this window period provides valuable insights into the recovery pattern and informs the decision to re-intervene which otherwise would not have been impossible. The developed system is not limited to analysis of patients with acute stroke alone. There are many parallel applications that can make use of the technology with the development of new algorithms. There are many similar systems demonstrated for stroke rehabilitation [14]. Similarly, gait analysis and age care devices can utilise the proposed system [16].

In future work, we plan to analyse the accelerometer signal using time frequency analysis in order to improve the classification of the index. As discussed earlier, the variation in the patient’s attitude (whether depressed or not) has considerable effect on the magnitude of hand movement which will impede the effectiveness of the algorithm. This can be addressed by reducing the impact of movement magnitude on the index by calculating better features. The efficiency of data collection also requires improvement by addressing the hardware issues such as power and radio frequency range. This can be done by developing a new hardware prototype dedicated to this objective, which will increase patient movement flexibility as well.

## Conclusion

Continuous monitoring of neurological activity is critical in effective stroke monitoring, particularly in the first few hours after the onset of stroke. It provides valuable insight into the recovery pattern and informs the decision to re-intervene with alternate or high dosage drugs. A proof-of-concept wireless accelerometer based motor activity monitoring system is presented in this paper. The architecture and the online algorithm have been presented and shown to work using 15 stroke patients with promising results. Norm based, signal magnitude area and average energy based features are compared for calculating a score equivalent to the NIHSS score and a high correlation is obtained using the energy based method. A new visualisation method is developed which helps the doctors to continuously monitor the recovery pattern. This lays the foundation for the development of a full fledged automated device for acute stroke monitoring.

## Competing interests

The authors declare that they have no competing interests.

## Authors’ contributions

JG, BY, MP conceived the study. JG carried out the hardware implementation, development of algorithms and drafted the manuscript. AR participated in the collection and analysis of the data with minor contribution in algorithm development. KF and BY are clinical experts who collected the data, recorded their observations. JG, BY and MP participated in the design and helped to draft the manuscript. All authors read and approved the final manuscript.
